# Rehabilitative monitoring of utricular dysfunction: VOCR findings before and after Epley maneuver in BPPV patients

**DOI:** 10.1007/s00405-025-09543-1

**Published:** 2025-07-08

**Authors:** Asya Fatma Men, Ahmet Adnan Cırık

**Affiliations:** 1 Department of Audiology, Faculty of Health Sciences, University of Health Sciences, Selimiye, Tıbbiye Cd No: 38, Üsküdar, Istanbul, Turkey; 2 Department of Otolaryngology, Umraniye Training and Research Hospital, University of Health Sciences, Umraniye, Istanbul, Turkey

**Keywords:** BPPV, Utricular dysfunction, vOCR, Epley maneuver, Otolith, Vestibular assessment

## Abstract

**Objective:**

This study aimed to objectively evaluate utricular dysfunction in patients with posterior canal benign paroxysmal positional vertigo (BPPV) using the video Ocular Counter-Roll (vOCR) test and to monitor functional changes following the Epley maneuver.

**Methods:**

The study included 30 patients diagnosed with BPPV (19 with right posterior canal involvement, 11 with left) and 30 age- and sex-matched healthy controls. After diagnosis was confirmed by the Dix-Hallpike test, all patients underwent the static vOCR test twice: before the Epley maneuver and during the follow-up examination in which nystagmus and vertigo were no longer observed in the Dix-Hallpike test. Measurements were performed using the Interacoustics VisualEyes™ 525 system and video oculography goggles. Participants were tested at 30° lateral body tilt positions, and only static torsional eye movements were analyzed.

**Results:**

Prior to treatment, vOCR values on the affected side were significantly lower compared to the unaffected side and the control group. After treatment, a marked improvement was observed in vOCR responses, and the differences between groups were no longer statistically significant. The mean vOCR value increased from 3.77° to 6.00°, and the asymmetry ratio decreased from 25.80 to 2.84%.

**Conclusion:**

The vOCR test appears to be an effective, rapid, and non-invasive clinical tool for evaluating utricular dysfunction and monitoring treatment response in BPPV patients. Its ability to provide objective data makes it a valuable complementary component in vestibular assessment protocols.

## Introduction

Benign Paroxysmal Positional Vertigo (BPPV), first described by Bárány in 1921, is one of the most common peripheral vestibular disorders characterized by vertigo attacks triggered by sudden head movements and lasting for seconds [1–3]. BPPV is associated with dysfunction of the utricle and sacculus, the otolith organs of the inner ear, and two theories are prominent in its pathophysiology. According to the widely accepted “cupulolithiasis” theory, otoconial crystals detach from the utricle macula and adhere to the cupula in the posterior semicircular canal, leading to symptoms [4]. Alternatively, the “canalithiasis” theory suggests that these crystals circulate freely in the perilymph, triggering symptoms [5, 6].

In the light of pathophysiologic theories, various clinical intervention methods have been developed for the relief of BPPV symptoms. In particular, the canalite reposition procedure (CRP), also known as the Epley maneuver, is a non-invasive and evidence-based approach that targets the displacement of autoconial particles from the posterior semicircular canal to the utricle [1, 4].

In the current literature, it is increasingly recognized that BPPV is not limited to the semicircular canals and that dysfunction of the otolithic system, especially of the utricle origin, plays an important role in the pathogenesis of the disease [7]. Studies show that utricular dysfunction is more common in BPPV patients than saccular dysfunction, and this is particularly evident in cases of recurrent BPPV [8].

vestibular evoked myogenic potential (VEMP) tests are one of the most widely used methods to evaluate utricular and saccule functions. Of these tests, cVEMP evaluates saccular function and oVEMP evaluates utricular function. In studies conducted in BPPV patients, oVEMP and cVEMP abnormalities have been observed. Especially oVEMP test is reported to be a reliable method in the detection of utricular lesions and objectively reflects the degree of dysfunction [9, 10]. Another method to evaluate utricular function is the video ocular counter-roll (vOCR) test, which has been included in the vestibular test battery by some manufacturers in recent years. This test, which stands out with its practicality, speed and economy in clinical practice, provides information about utricular function by analyzing the torsional eye movements that occur with lateral tilt of the head or body [11, 12].

The utricle is not only the source of autoconial crystals but also plays a key role in the regulation of torsional eye movements in response to head tilt movements [13, 14]. Therefore, impairments in utricular function may directly affect the severity and clinical course of BPPV symptoms [6, 8]. When the head is tilted from the ear to the shoulder, a compensatory torsional eye movement corresponding to the tilt of the head is generated by the function of the semicircular canals and otolith organs (especially the utricle). This vestibular reflex is the so-called ocular counter roll (OCR) response, which provides visual stability by sensing changes in head position [13, 14]. OCR consists of two components, static and dynamic. Dynamic OCR is characterized by a slow-phase torsional nystagmus in the opposite direction of the head tilt, with contribution from the semicircular canals and otolith organs. Static OCR is the response that originates from the utricle and maintains the torsional position of the eye when the head is tilted [12, 15].

The vOCR test has been shown to give reliable results in the assessment of utricular asymmetry both in healthy individuals [16] and in unilateral vestibular insufficiency, acute vestibular syndromes and vestibular neuritis [11, 17, 18]. Moreover, this method is valuable not only in detecting dysfunction but also in monitoring neurophysiological changes that occur during the rehabilitation process [11, 18]. The vOCR test assesses the vestibulo-ocular reflex by measuring the direction and amplitude of eye movements elicited by lateral head tilt.

However, to the best of the authors’ knowledge, there is no study in the literature that evaluates utricular responses with vOCR test after rehabilitative approaches that aim to redirect autoconia to the utricle, such as Epley maneuver. In this respect, vOCR stands out as a clinically valuable tool both in the diagnostic process and in the planning of individualized rehabilitation.

The aim of this study is to objectively evaluate utricular dysfunction in BPPV patients using vOCR measurements and to monitor functional improvement in the utricle after therapeutic maneuver. The findings are expected to make significant contributions to the literature in terms of the role of the vOCR test, which is still new in clinical practice, in the detection of utricular dysfunction and the traceability of neurophysiological changes occurring during the rehabilitation process.

## Materials and methods

### Ethical approval

The study was conducted in accordance with the Declaration of Helsinki and Good Clinical Practice (GCP) principles. The research protocol was approved by the Health Sciences University Scientific Research Ethics Committee (Meeting Date: 14.11.2024; Meeting Number: 2024/13; Decision Number: 13/35; Registration No: 24/689). Informed consent was obtained from all participants.

### Sample selection and participants

In this study, individuals diagnosed with posterior canal BPPV and healthy control subjects matched for age and gender were included from among students at the University of Health Sciences. A total of 60 individuals (30 BPPV patients and 30 healthy controls) participated in the study. In the BPPV group, 19 individuals had right posterior canal involvement and 11 individuals had left posterior canal involvement. The mean age of the participants was 49.67 ± 11.38 years, ranging from 30 to 65 years. G*Power (3.1.9.7) program was used to determine the sample size; it was calculated that at least 30 BPPV patients and 30 healthy individuals should participate in the study based on 5% significance level, 90% power and 0.5 effect size.

### Diagnosis and exclusion criteria

The diagnosis of posterior canal BPPV was established using the Dix-Hallpike [19], test, supported by other diagnostic positional tests such as the Supine Roll Test [20] and the Straight Head Hanging Maneuver [21], when necessary. During the Dix-Hallpike maneuver, the presence of positional nystagmus with a latency of 1–5 s, lasting 5–30 s, showing fatigue and torsional characteristics after head placement, was considered diagnostic. The nystagmus typically diminished when the patient was returned to the upright position. Only patients with idiopathic BPPV involving the posterior semicircular canal were included in the study. Individuals with clinical or laboratory findings suggestive of central nervous system disorders, ocular motility abnormalities, or middle ear pathology were excluded.

### Test procedure

In patients diagnosed with posterior canal BPPV, static vOCR measurements were performed using the vOCR method before canal reposition maneuver (Epley). Static vOCR was preferred in this study because dynamic vOCR tests are affected not only by the otolithic system but also by the semicircular canals. This may make it difficult to evaluate the otolith response in isolation, especially in canal-induced diseases such as BPPV. Static vOCR was preferred as a more specific and reliable method to assess the function of otolith organs only (especially the utricle). Epley maneuver was then performed. Patients were called back to the clinic for a follow-up appointment 7–10 days later. In the control examination, the Dix-Hallpike test was applied and vOCR measurements were performed again in individuals who were observed to have disappeared nystagmus and dizziness. All measurements were performed in the Audiology and Speech Disorders Laboratory of Ümraniye Training and Research Hospital in accordance with standard clinical protocols.

### Data collection tools

#### vOCR test

vOCR testing was performed using an Interacoustics VisualEyes™ 525 (Denmark) VNG device and Video Oculography (VOG) goggles. The device allows quantitative analysis of torsional eye movements with a standardized test protocol and automatic calibration procedure. During the test, participants were positioned in an upright sitting position on a stretcher and asked to focus on a 2.5 cm diameter fixed red target on a monitor 120 cm away. For vOCR measurements, a 30° lateral body tilt was used to improve participant comfort. An iPhone 11 with iOS 17.4.1 (Apple Inc., Cupertino, CA, USA) and a stabilizing phone bracket were used for precise body tilt adjustment [22]. A neck collar was worn to stabilize the participant’s neck during lateral body tilt. Before the start of the test, the VOG goggles were calibrated by recording iris patterns to ensure accurate tracking of eye movements.

**The implementation phases are as follows**:

- Participants focused on the target in an upright position for 30 seconds.
- In the following 30-second phases, a 30° lateral body tilt was applied in the right, center and left directions, respectively.
- At this time, the dynamic vOCR response was observed, followed by nystagmus recording as a static response.
- Only the static component was considered during the analyses, and three measurements were made for each position (right, left, center).
- One of the two audiologists kept the head position fixed, while the other operated the device.
- Static data taken between the 20th and 30th seconds were analyzed to reduce the influence of the dynamic response.
- Analyses were performed using the mean values obtained from the right and left eyes; the same tests were repeated at least one week later.

#### vOCR asymmetry ratio calculation

The vOCR asymmetry ratio was calculated as the ratio of the absolute difference between the measurements obtained from both sides to the absolute sum of the measurements. This calculation was used to assess the degree of imbalance between left and right OCR responses. An asymmetry ratio approaching 1 indicates a high level of asymmetry, while an asymmetry ratio approaching 0 indicates a low level of asymmetry [23].

### Epley maneuver

The Epley maneuver is a safe, evidence-based and non-invasive treatment approach that is widely used in patients with BPPV [24]. The Epley maneuver was performed twice, on the day of admission and one week later.

### Statistical method

IBM SPSS Statistics for Windows, Version 26.0 software was used to analyze the data obtained in the study. Mean ± standard deviation (SD), minimum-maximum values and interquartile range (IQR) were calculated as descriptive statistics for numerical data. Distributional characteristics of the data were evaluated by Shapiro-Wilk test. One-way analysis of variance (One-Way ANOVA) was used for group comparisons for normally distributed data. After ANOVA, Tukey HSD (Honestly Significant Difference) post-hoc test was applied to determine which pairs were responsible for the difference between the groups. Independent samples t-test was used for comparisons of two independent groups with normal distribution. Statistical significance level was accepted as *p* < 0.05.

In the healthy control group, there was no statistically significant difference between right and left lateral tilt vOCR values and asymmetry ratios (paired t-test, *p* = 0.707). Therefore, for consistency and accuracy of statistical analyses, the calculated mean vOCR value was used as a reference for all comparisons.

## Findings

### Demographic information

There are 19 participants in the right posterior canal involvement group. There were 11 participants in the left posterior canal involvement group. The mean age of the BPPV group was 49.43 ± 9.97 years (mean ± standard deviation). Gender distribution was 20 females and 10 males. There were 30 participants in the control group. The mean age of this group was 45.43 ± 7.87 years and the gender distribution was 23 females and 7 males. The distribution characteristics of the data were evaluated by Shapiro-Wilk test. Independent Samples t-test was used to compare variables with normal distribution and Mann-Whitney U test was used to compare variables without normal distribution (Tables 1 and 2).

**Table 1:** Statistical comparison of Pre- and Post-Epley vOCR values, group differences in BPPV patients and healthy controls

Comparison vOCR (o)	Mean ± SD	Min–Max	95% CI	IQR	*p*-value
Pre-Epley affected Pre-Epley unaffected	3.77 ± 1.03 5.99 ± 1.72	2.10–6.30 3.30–10.60	3.40–4.14 5.38–6.60	1.23 1.90	0.000
Post-Epley affected Post-Epley unaffected	6.00 ± 1.17 6.18 ± 1.36	3.10–9.30 3.30–9.00	5.58–6.42 5.70–6.67	1.15 1.07	0.519
Pre-Epley affected Control group	3.77 ± 1.03 6.35 ± 1.06	2.10–6.30 4.35–9.00	3.40–4.14 5.98–6.73	1.23 1.28	0.000
Pre-Epley unaffected Control group	5.99 ± 1.72 6.35 ± 1.06	3.30–10.60 4.35–9.00	5.38–6.60 5.98–6.73	1.90 1.28	0.127
Post-Epley affected Control group	6.00 ± 1.17 6.35 ± 1.06	3.10–9.30 4.35–9.00	5.58–6.42 5.98–6.73	1.15 1.28	0.166
Post-Epley unaffected Control group	6.18 ± 1.36 6.35 ± 1.06	3.30–9.00 4.35–9.00	5.70–6.67 5.98–6.73	1.07 1.28	0.588

There is a statistically significant difference between the Pre-Epley affected side, Pre-Epley unaffected side, and Control groups in terms of mean vOCR measurements. (One-Way ANOVA; F=35.624; *p*=0.0001<0.01). This difference was caused by the differences between the Pre-Epley affected side and Pre-Epley unaffected side (Unaffected> Affected; Tukey HSD test, *p*=0.0001<0.01), Pre-Epley affected side and Control group (Control> Affected; Tukey HSD test, *p*=0.0001<0.01), and Pre-Epley unaffected side and Control group (Control> Unaffected; Tukey HSD test, *p*=0.030<0.05)

There is no statistically significant difference between the Post-Epley affected side, Post-Epley unaffected side, and Control groups in terms of mean vOCR measurements. (One-Way ANOVA; F=0.657; *p*=0.521>0.05)

Comparisons using independent samples t-tests revealed:

No significant difference between the Post-Epley affected side and the Post-Epley unaffected side (*p*=0.585>0.05)

No significant difference between the Post-Epley affected side and the Control group (*p*=0.213>0.05)

No significant difference between the Post-Epley unaffected side and the Control group (*p*=.0.588>0.05)

**Table 2:** Comparison of vOCR asymmetry values before and after Epley maneuver and in the control group

Comparison vOCR (^o^)	Asymmetry %	*p*
Pre-Epley affected side- unaffected side	25.49	*p* <0.001^*^
Post-Epley affected side- unaffected side	2.83	*p* = 0.152^*^
Control group left lateral tilt- right lateral tilt	0.31	*p* = 0.118^**^

* Wilcoxon signed-rank, ** Mann-Whitney U

The asymmetry rates between the affected side and the unaffected side in the Pre-Epley period were compared by Wilcoxon signed-rank test and the results showed that there was a statistically significant difference between these two sides (*p* = 0.0000025; *p* <0.001). The mean asymmetry rate was calculated as 25.80% in this period.

After the Epley maneuver, the asymmetry rates observed between the affected and unaffected sides decreased significantly. This was evaluated by Wilcoxon signed-rank test and no statistically significant difference was found (*p* = 0.152; *p*> 0.05). The mean asymmetry rate after the maneuver was calculated as 2.84%.

**Fig. 1:**
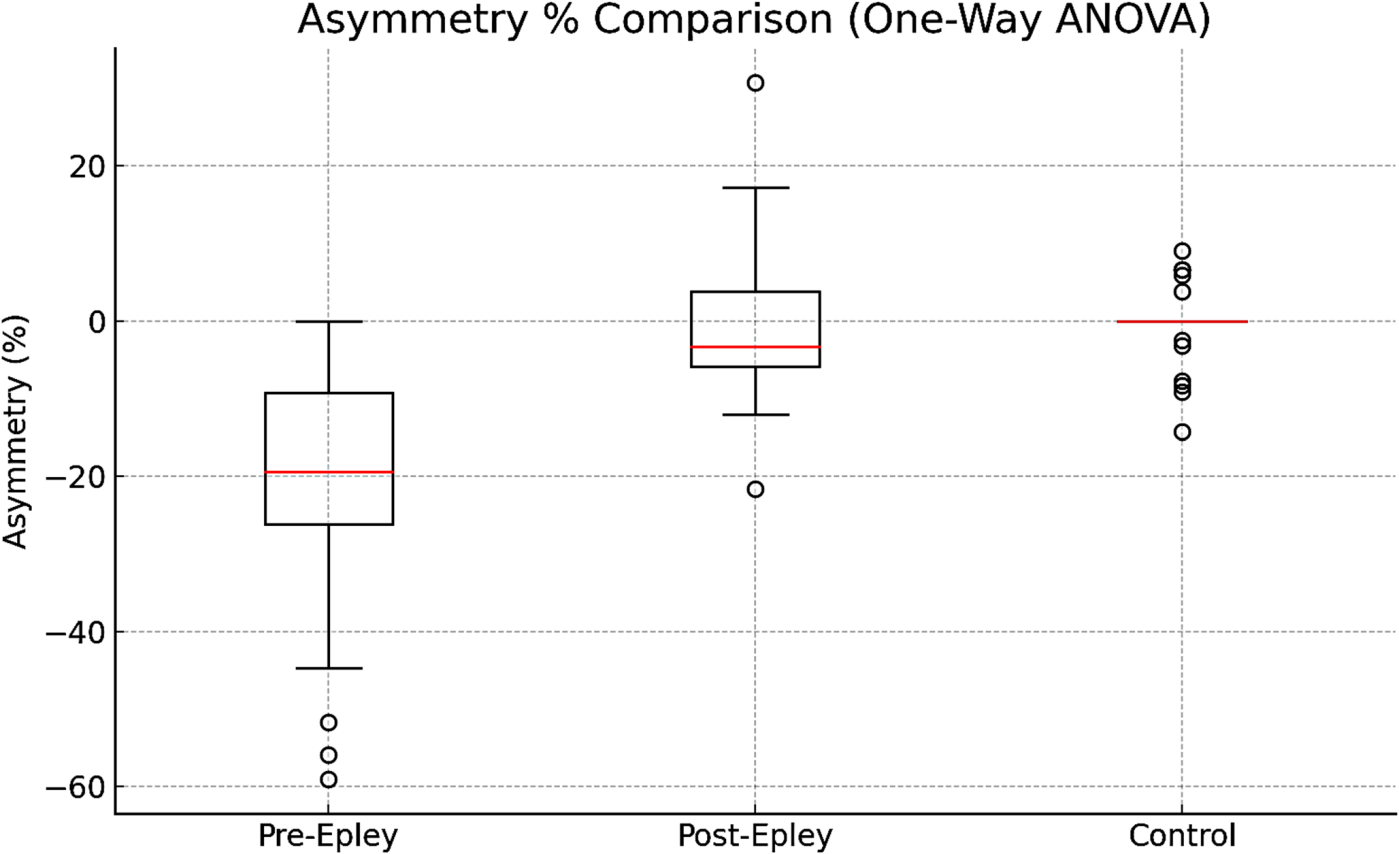
Comparative graph of vOCR asymmetry (%) distribution in BPPV patients before and after Epley maneuver and in the control group

**Fig. 2:**
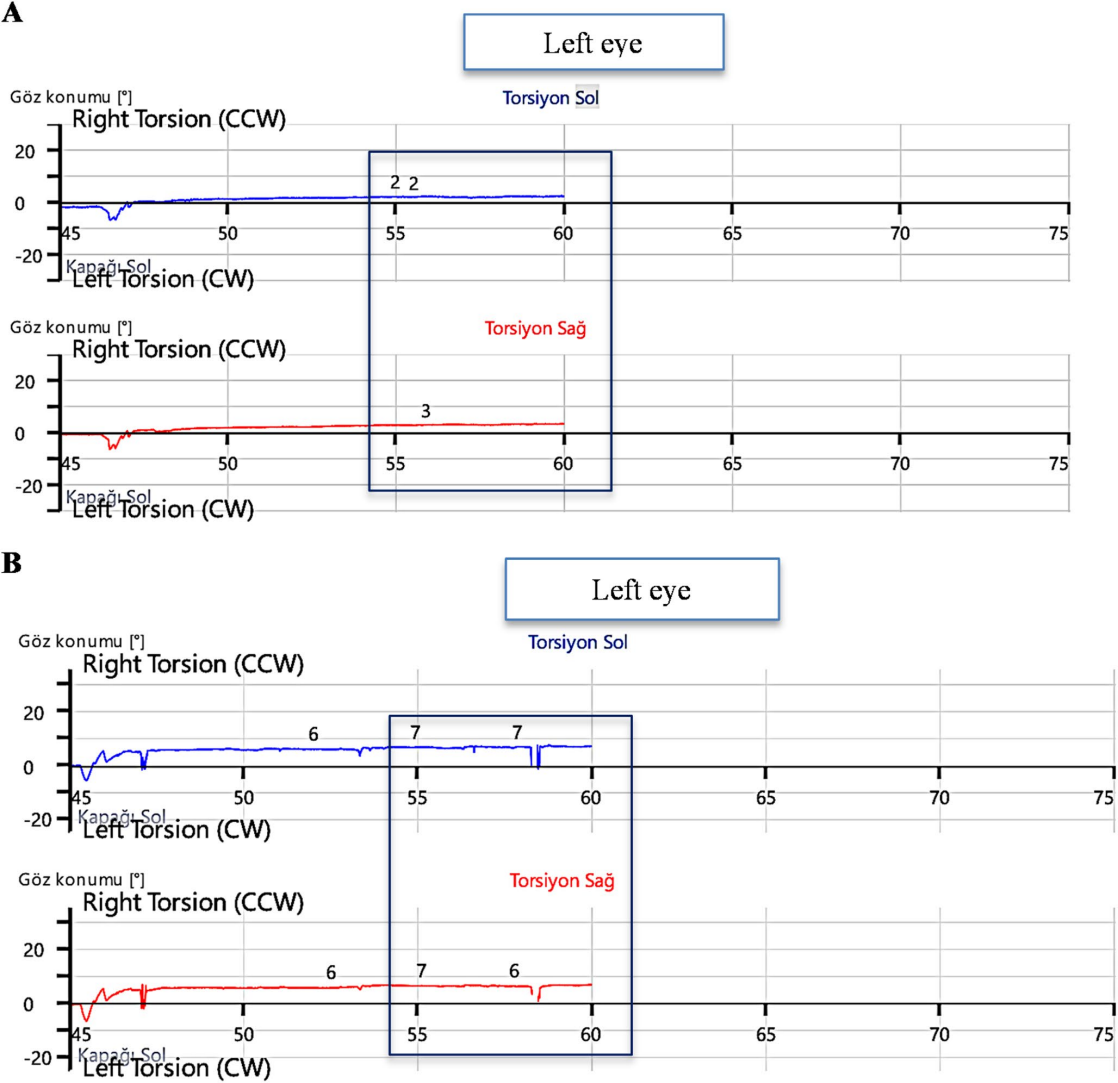
Left posterior canal BPPV Pre- Epley maneuver and Post- Epley maneuver vOCR findings. **A**: Pre-maneuver vOCR, **B**: post-maneuver vOCR

In addition, vOCR measurements between right and left lateral tilt in the control group were compared with the Mann-Whitney U test, and no statistically significant difference was found (*p* = 0.118; *p*> 0.05). The mean asymmetry rate between these groups was 1.81% (Figs. 1 and 2).

## Discussion

The aim of this study was to objectively assess utricular dysfunction in BPPV patients using vOCR measurements and to monitor functional improvement in the utricle after therapeutic maneuvering. Results showed that before the maneuver, vOCR values on the lesion side were significantly lower compared to both the unaffected side and healthy subjects. After the maneuver, a significant improvement in vOCR responses on the lesion side was observed. Moreover, the difference between BPPV and control group in terms of both lesion side and intact side vOCR values after treatment ceased to be statistically significant. In addition, the high asymmetry values observed between the lesion side and the healthy side before the treatment decreased significantly after the maneuver.

In patients with BPPV, displacement or degeneration of the otoconia in the utriculus and sacculus underlies the pathophysiologic process [8]. Therefore, tests evaluating the function of otolith organs are important for diagnosis and follow-up. VEMP tests (oVEMP and cVEMP) are frequently used for this purpose and show otolith dysfunction in BPPV patients with findings such as delayed latency, decreased amplitude and increased asymmetry [25, 26]. The vOCR test is a suitable alternative for clinical use due to its advantages such as non-invasiveness, rapid applicability, automatic analysis and integration into vestibular test batteries [12, 17]. In the literature, it is reported that vOCR has similar accuracy rates to VEMP tests in the evaluation of otolith functions [12, 17, 18].

In this study, low torsional responses and marked asymmetry were observed before the maneuver, and similar vOCR values were obtained after the maneuver on the lesion side, the unaffected side and the control group. Previous studies have reported that vOCR values below 4.5° in applications with 30° head tilt can identify patients with otolith dysfunction with 83% accuracy [17]. In our data, while the mean vOCR value was 3.77° on the lesion side before the maneuver, this value increased to 6° after the maneuver. The percentage of asymmetry decreased from 25.80 to 2.84%. This significant decrease shows that vOCR is effective not only in diagnosis but also in monitoring the response to treatment.

The Epley maneuver is one of the most commonly used repositioning methods in the treatment of BPPV and aims to return the posterior canal otoconia to the utricle with the help of gravity [24, 27]. In this study, the significant improvement in vOCR values after the maneuver suggests that the Epley maneuver not only provides symptomatic relief, but also leads to objective improvements in utricular function. This is important in terms of demonstrating the therapeutic efficacy of the Epley maneuver with objective parameters.

Studies in peripheral vestibular insufficiencies such as vestibular neuritis and bilateral vestibulopathy have also reported that vOCR is effective in revealing otolith dysfunction. Otero-Millan et al. (2017) reported significant vOCR decreases in patients with unilateral and bilateral vestibular loss [18]. Sadeghpour et al. (2021) showed significant response reductions in the acute phase in patients with vestibular loss [28]. My findings are consistent with these studies and a reduced degree of vOCR was observed on the lesion side.

The video head impulse test (vHIT) is a widely used test for the evaluation of the semicircular canals (SSC) [29–31]. However, it does not have sufficient sensitivity in the differential diagnosis in patients with a history of idiopathic BPPV [32–34]. vOCR offers the opportunity to examine otolith function with a focus on the utricle. Considering the role of utricular dysfunction in the pathophysiology of BPPV, the findings of this study suggest that vOCR may be a clinically meaningful tool to evaluate utricular function in BPPV. In this respect, the study provides pioneering findings that may guide clinical and scientific discussions on utricular dysfunction in BPPV.

Furthermore, a noteworthy feature of the vOCR test is its ability to assess tonic and regular afferent inputs from the utricular macula. This contrasts with ocular vestibular evoked myogenic potentials (oVEMPs), which primarily reflect irregular afferent function. Hence, vOCR and oVEMPs should not be regarded as alternative, but rather complementary methods for assessing utricular function [35]. This interplay is comparable to the relationship between caloric testing (low-frequency response) and the video head impulse test (vHIT), which measures high-frequency semicircular canal responses [36]. Integrating both vOCR and oVEMP into vestibular assessment protocols may enhance the understanding of otolithic integrity and help identify functional asymmetries more comprehensively.

## Conclusion

This study suggests that video vOCR test may be a clinically valuable tool in the evaluation of utricular function in BPPV patients, both in terms of diagnosis and monitoring response to treatment. Low vOCR values and high asymmetry were observed on the lesion side before Epley maneuver; after the maneuver, a significant improvement in vOCR responses and a significant decrease in asymmetry were found. These findings suggest that vOCR can be used not only to objectively detect otolith dysfunction, but also to monitor the efficacy of therapeutic maneuvers. The advantages of the vOCR test, such as its rapid applicability, non-invasiveness and integration into clinical systems, make it a complementary and functional tool in vestibular assessment protocols. Future studies with larger samples and long-term follow-up studies are expected to further consolidate the diagnostic power and clinical utility of vOCR.

## Limitations

This study has some limitations. First, the relatively small sample size limits the generalizability of the findings. Additionally, the study included only BPPV patients with posterior semicircular canal involvement, while other canal types were excluded. This limits the ability to comment on the diagnostic and monitoring value of the vOCR test in different BPPV variants. Furthermore, the tilt angle used in the vOCR test was kept constant, and responses at different angles were not compared. Early post-treatment vOCR measurements (e.g., within the first hour after the maneuver) were also not included in the study, which limits the understanding of immediate utricular responses following the treatment. In light of these limitations, future studies with larger samples, including different BPPV types and multiple post-treatment time points, are needed.
